# VEX1 Influences mVSG Expression During the Transition to Mammalian Infectivity in *Trypanosoma brucei*

**DOI:** 10.3389/fcell.2022.851475

**Published:** 2022-04-05

**Authors:** Eliane Tihon, Karinna Rubio-Peña, Annick Dujeancourt-Henry, Aline Crouzols, Brice Rotureau, Lucy Glover

**Affiliations:** ^1^ Trypanosome Molecular Biology, Institut Pasteur, Université Paris Cité, Paris, France; ^2^ Trypanosome Transmission Group, Trypanosome Cell Biology Unit, INSERM U1201 and, Institut Pasteur, Paris, France; ^3^ Parasitology Lab, Institut Pasteur of Guinea, Conakry, Guinea

**Keywords:** *Trypanosoma brucei*, VSG, antigenic variation, monoallelic expression, metacyclogenesis

## Abstract

The *Trypanosoma (T) brucei* life cycle alternates between the tsetse fly vector and the mammalian host. In the insect, *T. brucei* undergoes several developmental stages until it reaches the salivary gland and differentiates into the metacyclic form, which is capable of infecting the next mammalian host. Mammalian infectivity is dependent on expression of the metacyclic variant surface glycoprotein genes as the cells develop into mature metacyclics. The VEX complex is essential for monoallelic *variant surface glycoprotein* expression in *T. brucei* bloodstream form, however, initiation of expression of the surface proteins genes during metacyclic differentiation is poorly understood. To better understand the transition to mature metacyclics and the control of metacyclic *variant surface glycoprotein* expression we examined the role of VEX1 in this process. We show that modulating *VEX1* expression leads to a dysregulation of variant surface glycoprotein expression during metacyclogenesis, and that following both *in vivo* and *in vitro* metacyclic differentiation VEX1 relocalises from multiple nuclear foci in procyclic cells to one to two distinct nuclear foci in metacyclic cells - a pattern like the one seen in mammalian infective bloodstream forms. Our data suggest a role for VEX1 in the metacyclic differentiation process and their capacity to become infectious to the mammalian host.

## Introduction

The vector borne protozoan parasite *Trypanosoma* (*T*) *brucei* is the causative agent of Human African and Animal African Trypanosomiasis and remains today a pervasive public health issue in sub-Saharan Africa. Trypanosomes have a digenetic life cycle that transitions between the mammalian host and the tsetse fly (Glossinidae family) insect vector. During the life cycle, the trypanosome undergoes several important developmental transitions and includes the formation of up to 10 different morphological forms (Rotureau and Van Den Abbeele 2013) broadly grouped into trypomastigote and epimastigote morphotypes, and are defined by the relative positions of the nuclear and mitochondrial (kinetoplast) DNA in the cell (Hoare 1971).

Within the mammalian host, trypanosomes are covered in a dense layer of a single species of variant surface glycoprotein (VSG) and exist as proliferative slender forms or G_1_ arrested stumpy forms (Matthews 2005). Stumpy forms are pre-adapted to survival in the insect host and differentiate into procyclic forms, replacing the VSG coat with a procyclin coat following a tsetse fly blood meal. The trypanosomes then colonise the cardia, posterior midgut (Rose et al., 2020) and eventually migrate to the salivary glands (Van Den Abbeele et al., 1999; Sharma et al., 2008). The second major developmental transition in the tsetse fly is known as metacyclogenesis (Tetley et al., 1987; Rotureau et al., 2012), and occurs in the salivary glands. During this process epimastigote parasites that are attached to the salivary gland epithelium asymmetrically divide to produce pre-metacyclic cells that mature into mammalian infective metacyclic cells (Rotureau et al., 2012). Metacyclic trypanosomes acquire mammalian infectivity in the tsetse fly salivary gland where they begin to express a stage specific *metacyclic variant surface glycoprotein* (*mVSG*) gene (Tetley et al., 1987; Graham and Barry 1995). This mVSG coat is critical to the parasites ability to infect the mammalian host as it pre-adapts them for survival in the bloodstream (Vickerman 1969; Vickerman 1985). The life cycle is thus completed with the bite of a tsetse fly which deposits metacyclic trypanosomes into the mammalian host dermis (Caljon et al., 2016) where they differentiate and proliferate in the bloodstream and extracellular fluid and tissue (Capewell et al., 2016; Trindade et al., 2016). It is here, in the mammalian host, that the mVSG surface coat is replaced with a bloodstream form VSG coat (Stijlemans et al., 2016).

As with bloodstream form *VSG* expression, only one mVSG is present on the surface of the cell (Tetley et al., 1987; Ramey-Butler et al., 2015), and expression of *mVSG* genes transitions from multi-*mVSG* expression in pre-metacylics to singular m*VSG* gene expression in mature metacyclic cells as monoallelic expression is established (Hutchinson et al., 2021). *VSG* expression is tightly controlled, and *VSG* genes are transcribed from highly specialised loci know as expression sites. The metacyclic expression site (MES) share some similarity to the bloodstream form expression site (BES) in that they both: contain a single *VSG* gene, are found at telomeres and are transcribed by RNA Pol-1 (Barry et al., 1998; Ramey-Butler et al., 2015), and although MES and BES promoters are not conserved (Ginger et al., 2002), they are both recognised by the same CITFA Pol-I transcription factor (Kolev et al., 2017). However, while the BES is a polycistronic transcription unit composed of the *VSG* gene and several expression site associated genes (*ESAGS*), the MES is a monocistronic unit, harbouring only the *mVSG* gene (Alarcon et al., 1994). Very little is understood about the regulation of *mVSG* genes during the developmental transition to metacyclic form cells in the salivary gland of the tsetse fly. This is mostly due to the inability to culture quiescent and non-proliferative metacyclic cells that has hampered molecular studies on this life cycle stage. This has been overcome by ectopic over expression of RNA binding protein 6 (RBP6), in cultured procyclic cells which leads to the development of mammalian infective metacyclic forms (Kolev et al., 2012).

Several chromatin remodelling factors (Alsford et al., 2012), telomere binding proteins (Yang et al., 2009), the expression site body (ESB) (Navarro and Gull 2001), the inositol phosphate pathway (Cestari and Stuart 2015) and the histone chaperone CAF-1 (Faria et al., 2019) have all been implicated in the regulation and maintenance of bloodstream form *VSG* expression. The single active BES is also depleted of nucleosomes (Figueiredo and Cross 2010; Stanne and Rudenko 2010). This suggests control at the level of transcription, elongation and that chromatin reorganisation is critical for singular *VSG* expression. A novel chromatin protein, TbSAP is required for silencing *mVSG* genes in bloodstream form cells (Davies et al., 2021), and a targeted RNAi screen revealed 22 positive and negative regulators required for the developmental transitions towards mammalian infectivity (Toh et al., 2021). The VEX complex (Glover et al., 2016; Faria et al., 2019) is a monoallelic regulator that restricts *VSG* transcription to a single telomere, recruits the RNA splicing machinery to ensure high levels of processing (Faria et al., 2021) and is also required for silencing *mVSG* genes in bloodstream form parasites. The VEX complex is composed of VEX1, VEX2 (Glover et al., 2016; Faria et al., 2019), is enriched as one to two foci within the nucleus with one immediately adjacent to the ESB, or the site of active *VSG* expression, within a telomere cluster (Glover et al., 2016; Faria et al., 2019). Following *in vitro* differentiation, the VEX complex initially relocalises to the nuclear periphery, as has been reported for the active expression site (Landeira and Navarro 2007), but then redistributes within the nucleus. In the cultured procyclic insect stage cells, the VEX focus appears to be concomitant with all telomeres, suggesting that at least VEX1 differentially binds to telomeres in different life cycle stages (Glover et al., 2016), the purpose of this altered distribution remains unknown. Here we show that VEX1 is required for initiation of *mVSG* expression during metacyclogenesis and that VEX1 focal accumulation is life cycle stage-dependent.

## Materials and Methods

### *Trypanosoma brucei* Growth and Manipulation

Procyclic stage Trypanosoma brucei PT1 (Trenaman et al., 2019) cells were grown in SDM-79 medium at 27°C. Cell density was determined using a haemocytometer. For transformation, 2 × 10^7^ cells were spun for 10 min at 1,000 g at room temperature the supernatant discarded and washed in 2 ml of prewarmed cytomix and spun as before. The cell pellet was resuspended in prewarmed 100 µL cytomix solution (van den Hoff et al., 1992) with 10 µg linearised DNA and placed in a 0.2 cm gap cuvette, and nucleofected (Lonza) using the X-014 program. The transfected cells were placed into 10 ml SDM-79 medium only and placed in an incubator to allow the cells to recover overnight. Serial dilutions plated out into 96 well plates at a 1:25, 1:50, and neat. 1 × 10^6^/ml wild type or untransformed cells were added to the dilutions to condition the medium. Antat 1.1E (EATRO1125) cells were grown in HMI-11 medium at 37.4°C with 5% CO_2_ and the density of cell cultures measured using a haemocytometer keeping cells bellow 1 × 10^5^ cells/ml. Transformation of cell lines was carried out by centrifuging 2.5 × 10^7^ cells at 1,000 g for 10 min at room temperature. The cell pellet was resuspended with 10 μg linearized DNA in 100 μL of warm cytomix solution (van den Hoff et al., 1992), placed in a cuvette (0.2 cm gap) and transformed using a Nucleofector™ (Lonza) (X-001 function). Transfected cells were recovered in 36 ml of warm HMI-11 at 37°C for 4–6 h, after which cells were plated out in 48 well plates at 1:5, 1:10, 1:50 and neat serial dilutions with the required drug selection. For metacyclic differentiation induction, the PT1 RBP6 overexpression cell line was grown in SDM-80 medium with 10% heat inactivated FBS, without glucose and 50 mM N-acetyl glucose-amine to block uptake of residual glucose molecules from the FBS (Dolezelova et al., 2020). RBP6 overexpression was induced with 10 μg/ml of tetracycline and the cell line maintained between 2–5 × 10^6^ cells/mL—exponential mid-log growth phase. Hygromycin and Blasticidin were selected at 2 μg ml^−1^, 2.5 μg ml^−1^, and 10 μg ml^−1^ respectively. Puromycin, phleomycin, hygromycin, blasticidin, and tetracycline were maintained at 1 μg ml^−1^.

### Cell Line Set Up

To construct PT1 RBP6 overexpression cell line, RBP6 was amplified from wild type genomic DNA using primers RBP6F and RBP6R (See Table 1 for sequence) and cloned into pRPa vector (Alsford et al., 2005) using HindIII—BamH1. The resulting construct was linearized with Asc1. To construct the constitutive VEX1 overexpression construct pRPΔopVEX1, we digested pRPaVEX1 ^i6m^ and pRPΔop with HindIII—BamH1 and ligated. pRPaVEX1^i6m^, pRPaVEX1^isl^ and pNATVEX1^12myc^ are described in (Glover et al., 2016).

**TABLE 1:** Primers used in this study.

Cloning	
RBP6 Forward	GAT​CAA​GCT​TAT​GTT​CTA​CCC​CAA​CAG​CCC​G
RBP6 Reverse	GAT​CGG​ATC​CTC​AAC​CAG​CGG​CAC​CG
**qPCR**	
Actin F	GTA​CCA​CTG​GCA​TTG​TTC​TCG
Actin R	CTT​CAT​GAG​ATA​TTC​CGT​CAG​GTC
VEX1-4 F	ACG​ACC​GAA​GTT​GTT​TGG​GT
VEX1-4 R	TAA​CCT​TCT​GCT​GCT​GAC​CG
RBP6-4 F	TTT​TGC​CAT​GCG​GAA​GAT​GC
RBP6-4 R	GGG​AAC​CCG​CAT​GAA​CGT​AT
mVSG 397 Forward	TGA​AGC​TGT​GAA​AGG​GAC​AG
mVSG 397 Reverse	GAG​GGC​GAA​TTG​TTT​GTT​TAG​G
mVSG 531 Forward	GAC​GAA​AGC​CTG​GGT​AAC​ATA​AA
mVSG 531 Reverse	CCG​CAG​CTC​GTT​GAT​AGT​ATT​G
mVSG 639 Forward	CCG​ACG​ATG​AAC​ACA​GTT​GA
mVSG 639 Reverse	TCT​ATG​CCG​TTC​GCC​TTT​AC
mVSG 653 Forward	GGG​CTG​TTT​CGC​GAC​TAA​TA
mVSG 653 Reverse	CGT​GGT​GAA​GTC​TCC​TGT​TT
mVSG 1954 Forward	GCA​GAG​GCC​TTA​GCA​CTA​AAT
mVSG 1954 Reverse	GGA​GTT​GAC​TTT​CCT​CCA​TCA​G
mVSG559 F1	CAG​AGC​AAA​CCA​GGC​GCT​G
mVSG559 R1	GTGTGTCCGCTGCAGTCG
mVSG559 F2	ACT​GCC​TGA​GCT​AAA​GGC​AGA
mVSG559 R2	ACC​GCG​TAG​CCG​TTA​GTG​TG
mVSG636 F1	ACG​TTG​GCA​GCC​AAT​GCA​G
mVSG636 R1	AAGCTGCGCTACACCGTC
mVSG636 F2	ATC​AGC​CAT​CGG​CGA​ACA​AG
mVSG636 R2	CCA​GCA​ACG​TGC​TAG​CTG​C
mVSG3591 F1	TTA​ACG​GCG​GCG​ACT​GGC​A
mVSG3591 R1	CGC​TGG​GCT​GCC​TTG​ACA​A
mVSG3591 F2	AGC​GAC​GAT​GGA​GCC​GTA​A
mVSG3591 R2	CTT​GCT​TTG​GCT​GCC​TGT​G

### Immunofluorescence Microscopy

Immunofluorescence analysis was carried out using standard protocols as described previously (Glover et al., 2013). Rabbit α-myc (Cell Signalling, # 71D10) (1: 200) and rabbit α- CRD (1: 500) [Davids-Biotechnologies, (Zamze et al., 1988)]. Secondary anti-sera used were goat α-rabbit AlexaFlour^®^ 555, goat α-rabbit AlexaFlour^®^ 488 (1:1,000). Samples were mounted in Vectashield (Vector Laboratories) containing 4, 6-diamidino-2-phenylindole (DAPI). In *T. brucei*, DAPI-stained nuclear and mitochondrial DNA can be used as cytological markers (Woodward and Gull 1990); Images were captured using a ZEISS Imager 72 epifluorescence microscope with an Axiocam 506 mono camera and images were processed in ImageJ.

### RNA Analysis

RNA samples were taken at 0-, 4- and 8-days post RBP6 and VEX1 overexpression or knockdown induction. RNA was extracted from 50 ml of culture at 2 × 10^6^ cells/ml. RNA-seq was carried out on a BGISeq platform at The Beijing Genome Institute (BGI). Reads were mapped to a hybrid genome assembly consisting of the T. brucei 427 reference genome plus the bloodstream VSG-ESs (Hertz-Fowler et al., 2008; Cross et al., 2014; Muller et al., 2018). Bowtie 2-mapping was used with the parameters --very-sensitive --no-discordant --phred33. Alignment files were manipulated with SAMtools (Li et al., 2009). Per-gene read counts were derived using the Artemis genome browser (Carver et al., 2012); MapQ, 0. Read counts were normalised using edgeR and differential expression was determined with classic edgeR. RPKM values were derived from normalised read counts in edgeR (Robinson et al., 2010).

### qPCR and RT-qPCR Analysis

The expression levels of *RBP6*, *VEX1*, and *mVSG* genes was analysed by RT-qPCR using Luna Universal qPCR MasterMix (NEB) with 500 nM of primers. All primer pairs are listed in Table 1. RNA was extracted using a Qiagen RNeasy Kit and the samples were treated with DNase 1 for 1 h according to manufactures instructions and eluted in 30 µL of RNase free water. The samples were quantified using a Nanodrop (ThermoFisher). cDNA was prepared using SuperScript IV (ThermoFisher) following the supplier instructions from 1–2 µg RNA with a polyT primer. For each pair of primers (used at 500 nM), triplicates of each sample were run per plate (Hard-shell PCR Plates 96 well, thin wall; Bio-Rad), which were sealed with Microseal “B” Seals (BioRad). All experiments were run on a CFX96 Touch Real-time Detection system with a C1000 Touch Thermal cycler (Bio-Rad), using the following PCR cycling conditions: 95°C for 1 min, followed by 40 cycles of 95°C for 15 s and 60°C for 30 s (fluorescence intensity data collected at the end of the last step). Data was then analysed by relative quantification using the ΔΔCt method (CFX Maestro software—Bio-Rad) and Cq determination regression was used. In all cases, product abundance was determined relative to an actin control locus.

### Fly Infections

Tsetse flies (*Glossina morsitans*) were maintained at 27°C and 70% hygrometry in Roubaud cages, in groups of 50 male flies per cage. Pleomorphic trypanosome cell lines were maintained at 1 × 10^5^ cells/ml density in HMI-9 medium plus 10% FBS at 37°C with 5% CO_2_. *In vitro* stumpy differentiation was induced in HMI-9, supplemented with 10% FBS without antibiotics, by adding 8-pCPT-2′-O-Me-5′-AMP (5 μM) (BioLog-Life Science Institut) to the culture 48 h before fly infection (Laxman et al., 2006). On the day of infection, trypanosomes were resuspended at 10^6^ cells per ml in SDM79 with no antibiotics supplemented with 10 mM glutathione prior infection (MacLeod et al., 2007). Flies were fed on infected media through a silicone membrane and maintained until dissection by feeding three times per week on sheep’s blood in heparin. Flies were starved for 2 days before dissection at day 28. Imaging was carried out using a ZEISS Imager 72 epifluorescence microscope with an Axiocam 506 mono camera. Single images (for DIC) or multichannel stacks (for fluorescence) of images every 0.24 µm were acquired. When maximum intensity Z-projections are presented, they were generated using Fiji (Schlindelin et al., 2012).

## Results

### Loss of VEX1 Results in *mVSG* Expression in Insect Stage Cells

**FIGURE 1:**
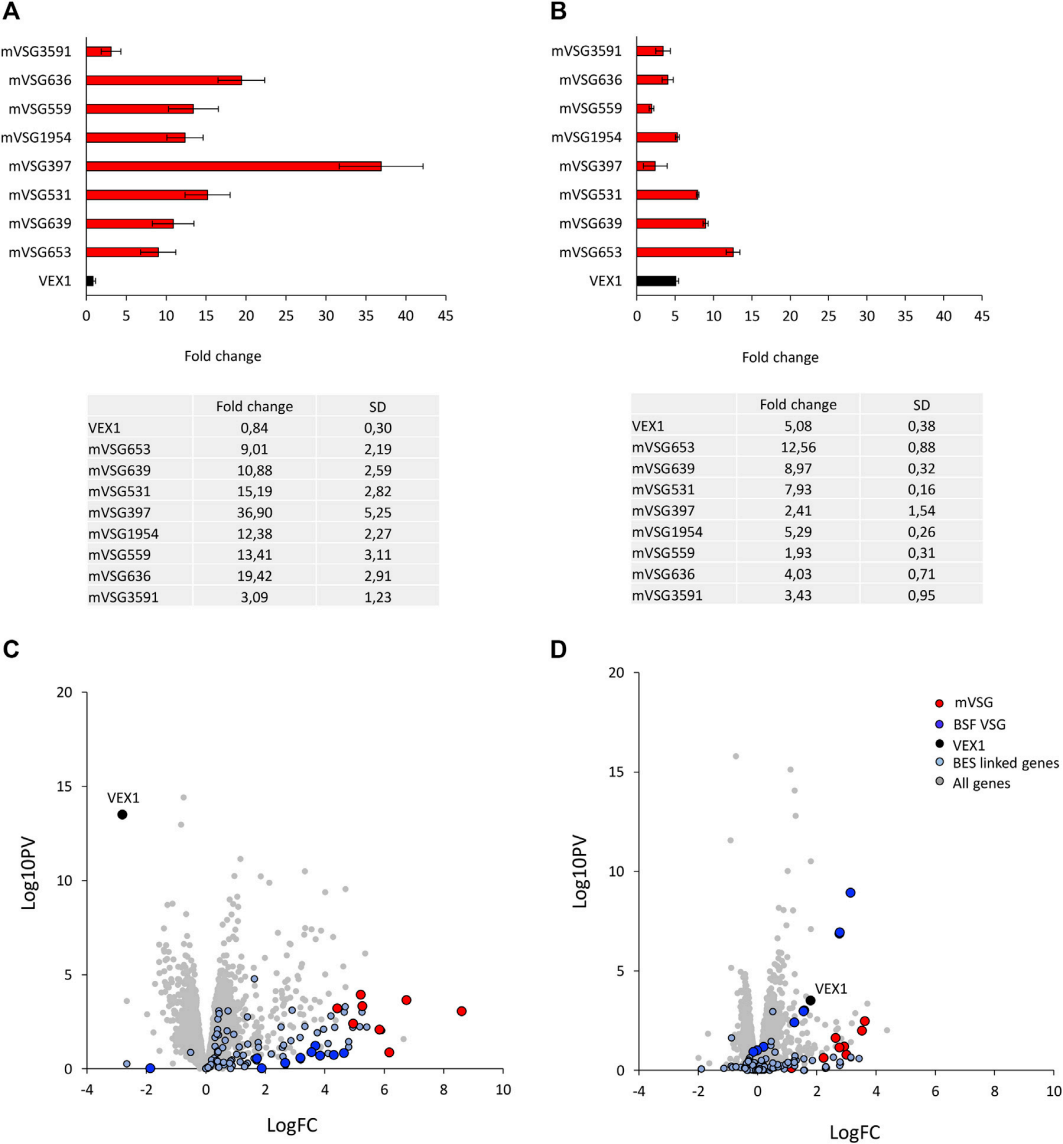
VEX1 depletion results in upregulation of metacyclic VSG expression sites in insect stage cells. **(A)** qRT-PCR of 8 mVSGs and VEX1 are shown as fold-change relative to wild-type cells, *N* = 3 (RNAi) and *N* = 4 (overexpression) for biological replicates, error bars denote SD. and table with fold change and SD values below for **(A)** VEX1 RNAi (96 h) or **(B)** VEX1 overexpression (96 h). RNA-seq analysis following **(C)** VEX1 RNAi or **(D)** overexpression for 96 h. Values are averages from three independent biological replicates relative to wild-type controls. Red circles, mVSG genes; blue circles, BES VSG genes; black circle, VEX1; grey circles, all genes.

The transition between developmental stages is accompanied by changes in gene expression, including the silencing or activation of both the BESs and MESs. The bloodstream form to procyclic stage differentiation is marked by the replacement of the VSG surface coat with EP and GPEET procyclins (Acosta-Serrano et al., 2001). Given the VEX complex association with the *VSG* transcription compartment in the bloodstream form cells and that it redistributes upon differentiation (Glover et al., 2016; Faria et al., 2019; Faria et al., 2021), we wanted to ask whether VEX1 was required for silencing BES and MESs in the procyclic stage cells. We first assessed expression of the 8 *mVSG* genes (Muller et al., 2018) and *VEX1* by RT-qPCR in *VEX1* overexpression or knock down backgrounds (Figures 1A,B). *VEX1* RNAi led to a greater derepression of the 8 *mVSG* genes (Figure 1A; Supplementary Figure S1A) compared to *VEX1* overexpression (Figure 1B; Supplementary Figure S1B)—average fold change of 15,04 compared to 5,82 respectively. Given this striking difference between expression of the *mVSG* genes according to the VEX1 expression level, we wanted to determine whether this was similar for the BES *VSGs*. Transcriptomic analysis of *VEX1* silenced by RNAi or overexpressed for 96 h showed similar patterns to the RT-qPCR, where RNAi led to a greater derepression of the 8 *mVSG* genes than overexpression, suggesting a primarily silencing function for VEX1 in procyclic stage cells. When we analysed BES linked genes, we found that overall, there was a subtler derepression as compared to the *mVSG* genes (Figures 1C,D), but this was more pronounced following *VEX1* RNAi, again suggesting that VEX1 is primarily required for silencing in this life cycle stage. Our data point to VEX1 having a role as a negative regulator of *VSG* expression in procyclic stage cells, where knocking down expression of *VEX1* from the cell has a stronger effect compared to overexpression. This suggests that VEX1 is required for efficient silencing of *mVSG* ES promoters in procyclic stage cells.

### VEX1 Focal Accumulation Is Life Cycle Stage Dependent

**FIGURE 2:**
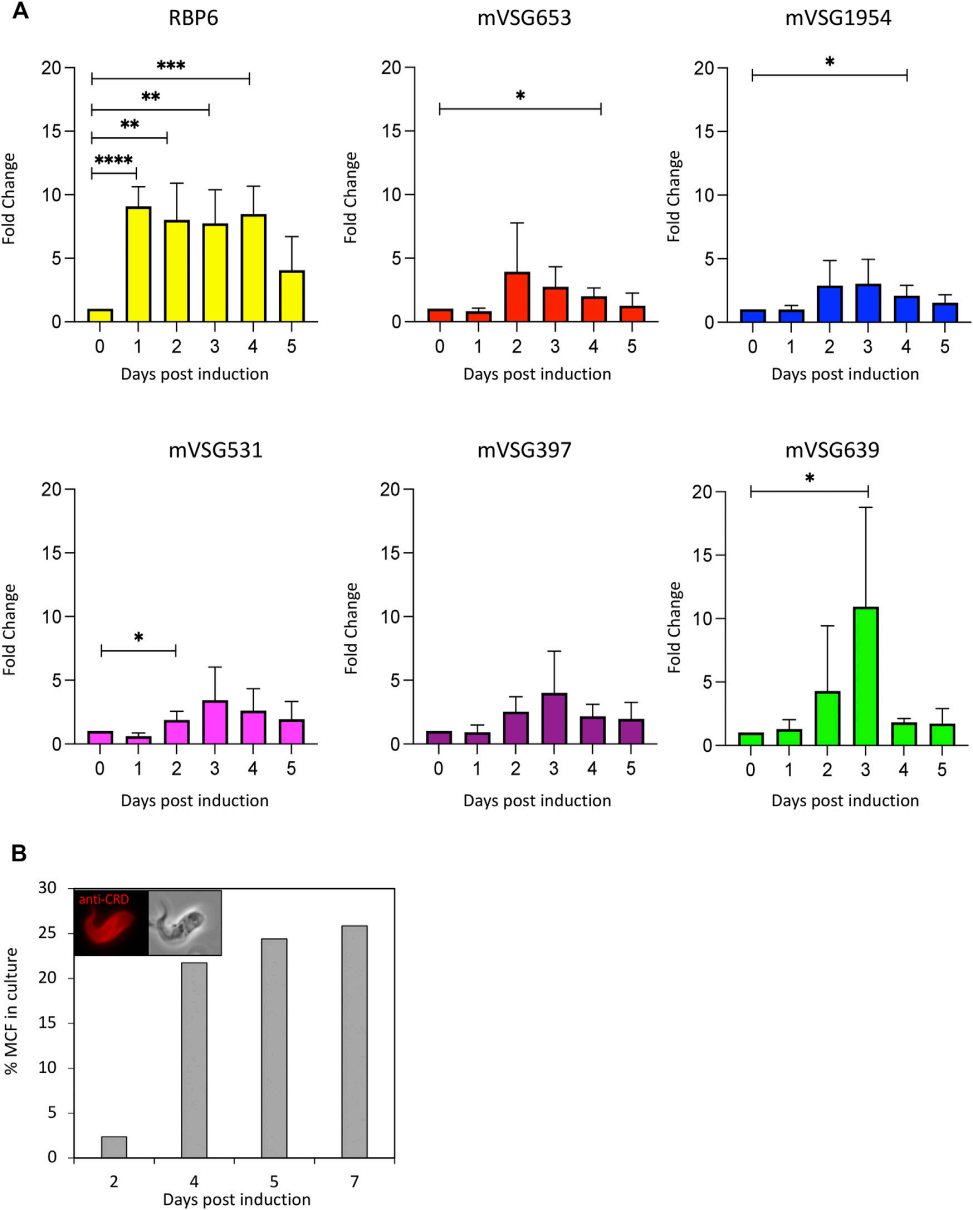
*In vitro* induction of metacyclogenesis. **(A)** Left panel: RT-qPCR shows fold change relative to wild-type of RBP6 and mVSG expression over 5 days. *N* = 3 for biological replicates, error bars denote SD. Significance calculated using student t test (*, *p*—0.02; **, *p*—0.05; ***, *p*—0.001; ****, *p*—0.0001; *p*—< 0.0001) **(B)** Quantification of the number of metacyclic cells following RBP6 induction using anti-CRD antibody, morphology and the position of the nucleus and kinetoplast.

**FIGURE 3:**
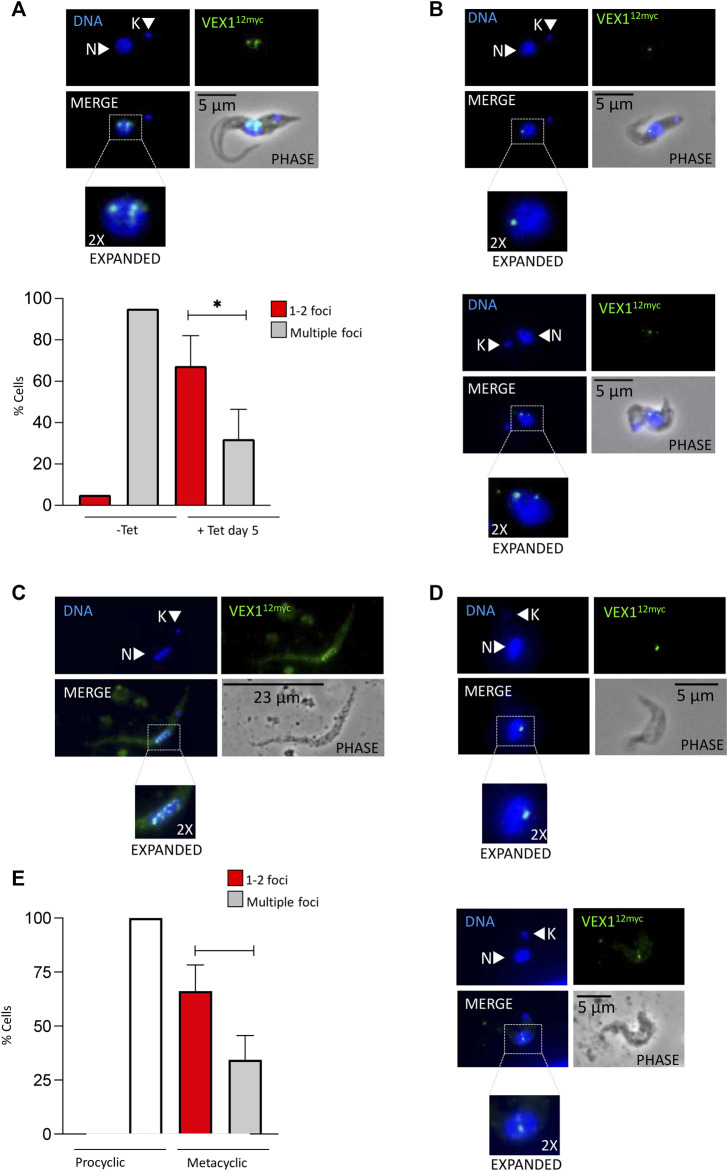
VEX1 nuclear distribution is life-cycle specific. Immunofluorescence analysis of VEX1 localization **(A)** Upper panel: RBP6 uninduced culture derived procyclic cells. Lower Panel: Quantification of nuclear VEX1 foci Uninduced and day 5 induced RBP6 cultured procyclic and metacyclic cells (*n* ≥ 100). Non dividing cells were counted, morphology and the position of the nucleus and kinetoplast were used to count only procyclic cells for uninduced. The images correspond to maximal 3D projections of 0.24 μm stacks; scale bars 23 or 5 μm. Error bar, SD. Significance calculated using student t test (*, *p*—0.04) **(B)** RBP6 induced culture derived metacyclic cells with either one or two foci **(C)** tsetse fly midgut derived late procyclic/meosocyclic cells **(D)** tsetse fly salivary gland derived metacyclic cells with either one or two VEX1 foci **(E)** Quantification of nuclear VEX1 foci in tsetse fly derived procyclic and metacyclic cells (*n* ≥ 100). The images correspond to maximal 3D projections of 0.24 μm stacks; scale bars 23 or 5 μm. Error bar, SD. Counts from 12 dissected tsetse flies. N, nucleus; K, kinetoplast. Significance calculated using student t test (*, *p*—0.02).

**FIGURE 4:**
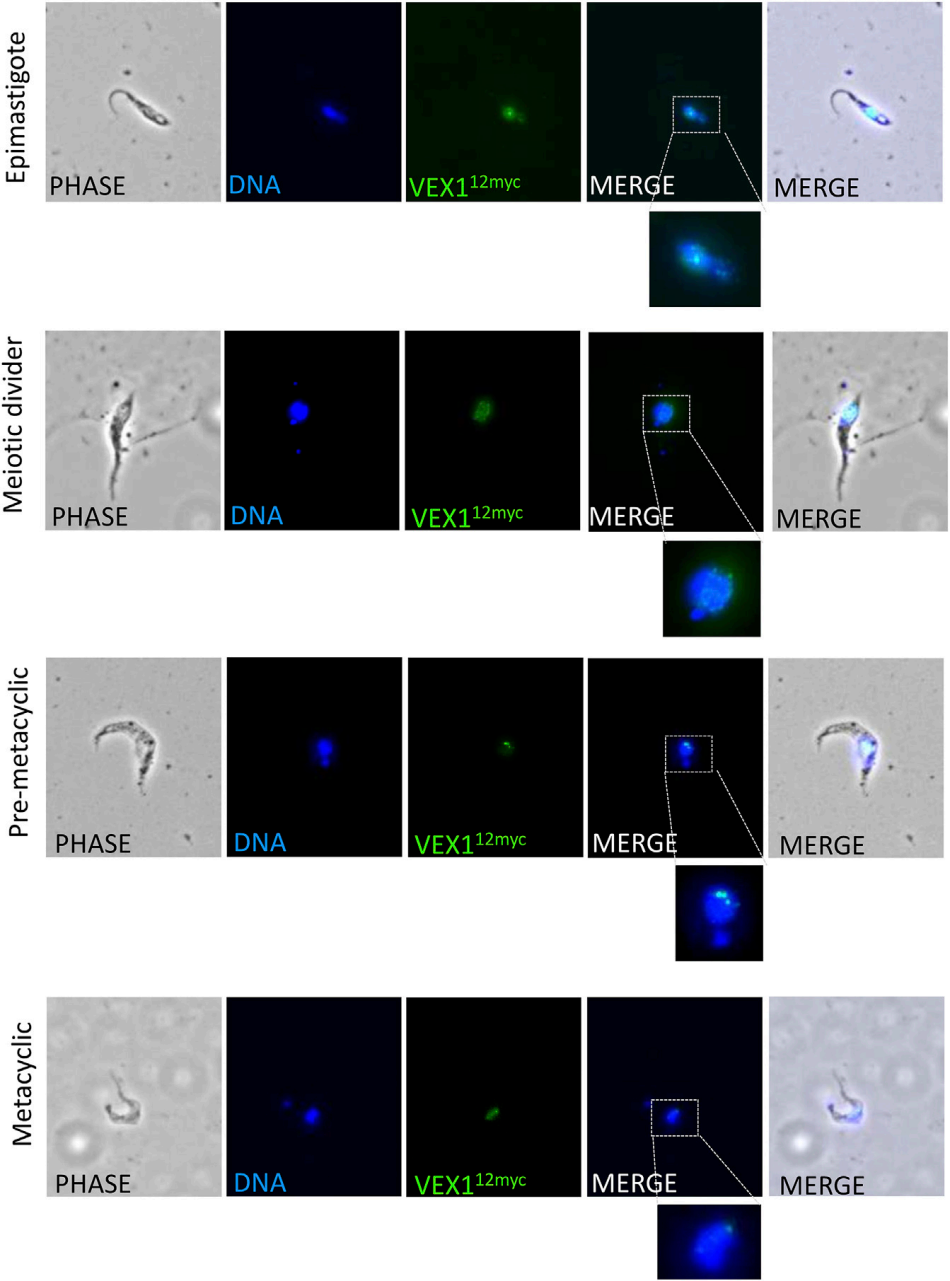
VEX1 localization in Tsetse fly derived cells. Immunofluorescence analysis of tsetse fly derived trypanosome cells. The images correspond to maximal 3D projections of 0.24 μm stacks; scale bars 5 μm. N, nucleus; K, kinetoplast.

As we have shown VEX1 is required for efficient silencing of expression-site linked *VSG* genes in insect stage cells, we then asked whether the distribution of VEX1 in the nucleus changed depending on the life cycle stage in the tsetse fly, and specifically in metacyclic cells where a *VSG* is expressed. To assess VEX1 localisation in metacyclic cells, we used the inducible *RBP6* expression system (Kolev et al., 2012). In this system, metacyclic cells are produced spontaneously rather than in a temporal order (Kolev et al., 2012; Ramey-Butler et al., 2015), allowing us to capture both early stage and mature metacyclic cells. We found, as previously shown, that induction of RBP6 stimulates metacyclogenesis *in vitro* and leads to the expression of *mVSGs* and the production of metacyclic cells in culture (Figure 2A). We assessed the expression of 5 *mVSG* genes and *RBP6* by RT-qPCR and found a significant increase in *mVSG653*, *mVSG1954*, *mVSG531* and *mVSG639* expression over day 2 to day 4 (Figure 2A; Table 2). We then scored the number of metacyclic cells in culture using a pan-VSG antibody (anti-CRD). Between day 5 and 7 up to 25% of the culture were metacyclic cells (Figure 2B). Following *in vitro* differentiation to insect stage cells, the active BES relocalises to the nuclear periphery (Landeira and Navarro 2007), while the VEX complex is redistributed from one—two nuclear foci to a multifocal distribution that appears to be concomitant with all telomeres (Glover et al., 2016). This differentiation step is also associated with a substantial increase in protein abundance VEX2 (Faria et al., 2019). A similar pattern was observed in the uninduced procyclic stage cells (Figure 3A), where VEX1 forms a multi-focal pattern in the nucleus. Following 5 days of *RBP6* overexpression, metacyclic cells were identified in culture based on cell morphology and the position of the nucleus and kinetoplast. We found that in these metacyclic cells there were significantly more VEX1 forming one to two foci (Figures 3A,B; Procyclic cells 95% formed multiple foci versus 68% of metacyclic cells with 1-2 foci), suggesting that the pattern of VEX1 accumulation in the nucleus is life cycle stage specific. We then wanted to confirm these findings *in vivo*. Tsetse flies (Glossina morsitans morsitans) were infected with Antat 1.1E bloodstream form cells with natively tagged VEX1. As was seen in the *in vitro* RBP6 system, VEX1 formed a multi focal pattern in midgut trypomastigote cells (procyclic and mesocyclic forms, Figures 3C,E; Supplementary Figure S2). Approximately 60% of metacyclic cells had one to two foci (Figures 3D,E; Supplementary Figure S3), and in approximately 40% we could detect 3 or more VEX1 foci, these may be early metacyclic where monoallelic *mVSG* expression is not yet established (Hutchinson et al., 2021). We also examined additional life cycle stages found throughout in the tsetse fly and found that VEX1 was distributed across the nucleus in a multi-focal pattern. Therefore, in metacyclic cells one to two VEX1 foci per nucleus was the dominated pattern (Figure 4; Supplementary Figure S3). We, therefore, see a similar pattern of VEX1 distribution both *in vitro* and *in vivo.* The VEX1 localisation we see in metacyclic is reminiscent of that seen in bloodstream form cells (Glover et al., 2016; Faria et al., 2019; Faria et al., 2021) where the VEX complex is required for singular *VSG* expression.

**TABLE 2:** RT-qPCR values for RBP6, VEX1 and mVSG expression Fold change and SD values N = 3 (Parental), N = 4 (overexpression) and N = 2 (RNAi) for biological replicates.

	RBP6		mVSG653		mVSG1954		mVSG531		mVSG639		mVSG397	
	Fold change	SD	Fold change	SD	Fold change	SD	Fold change	SD	Fold change	SD	Fold change	SD
D0	1	0	1	0	1	0	1	0	1	0	1	0
D1	9.08	1.55	0.83	0.24	1.00	0.32	0.61	0.27	1.30	0.74	0.90	0.58
D2	8.01	2.89	3.93	3.85	2.90	1.96	1.90	0.67	4.30	5.14	2.55	1.16
D3	7.74	2.65	2.74	1.59	3.04	1.92	3.43	2.60	10.94	7.84	4.01	3.28
D4	8.47	2.20	1.99	0.68	2.09	0.83	2.63	1.70	9.26	14.87	2.17	0.95
D5	4.07	2.64	1.26	0.99	1.54	0.64	1.95	1.39	1.73	1.18	1.98	1.29

### VEX1 Modulates *mVSG* Expression During Metacyclogenesis

**FIGURE 5:**
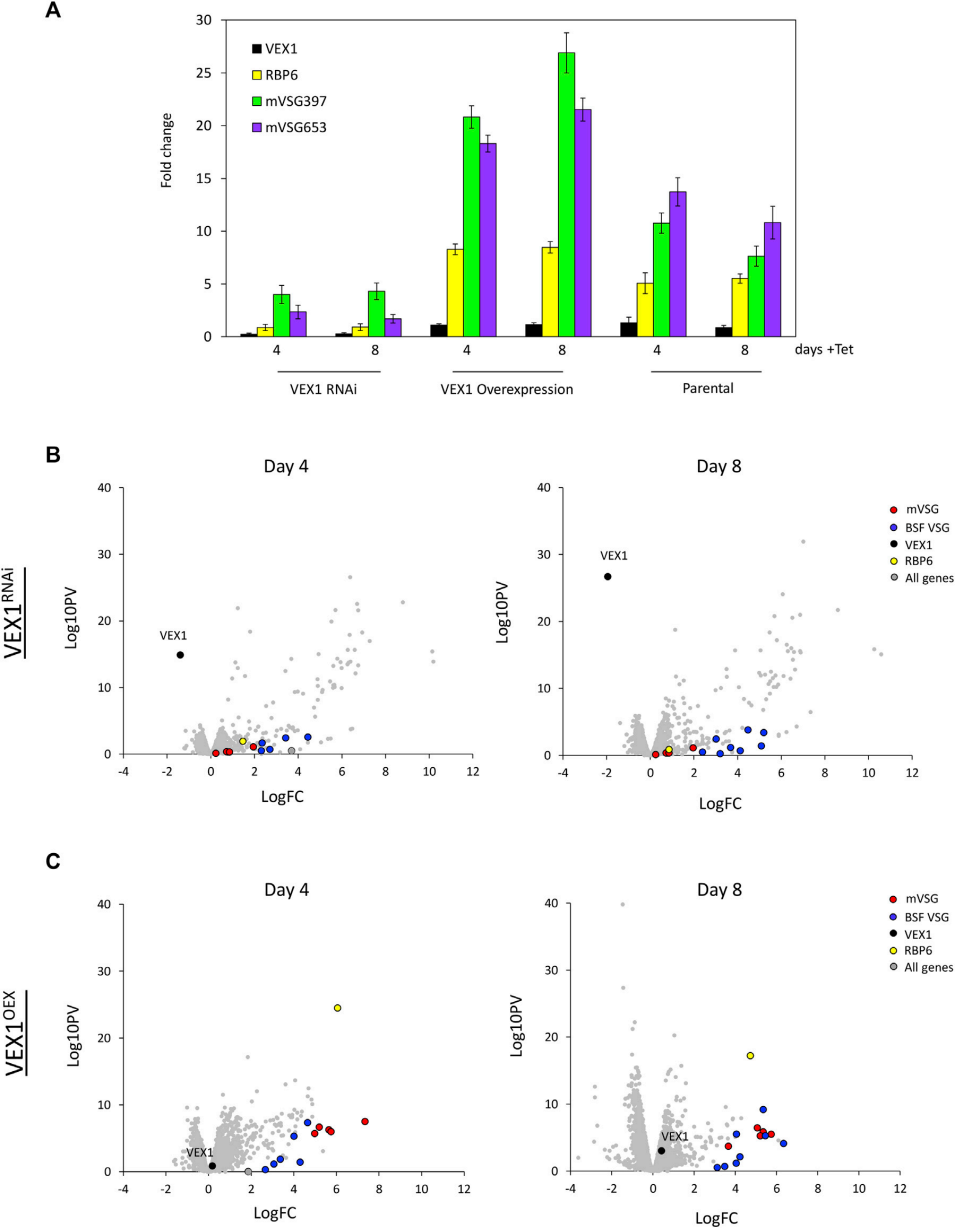
VEX1 is required for metacyclogenesis. **(A)** qRT-PCR of two mVSGs genes, VEX1 and RBP6 are shown at 96 and 192 h post RBP6 induction with either VEX1 RNAi or overexpression, as fold-change relative to uninduced cells. *N* = 3 (Parental), *N* = 4 (overexpression) and *N* = 2 (RNAi) for biological replicates, Error bars denote SD. **(B)** RNA-seq analysis following RBP6 induction and VEX1 RNAi at 4- and 8-days post induction, values are averages from three independent RNAi strains relative to wild-type controls. **(C)** RNA-seq analysis following RBP6 induction and VEX1 overexpression at 4- and 8-days post induction, values are averages from three independent VEX1 overexpression strains relative to wild-type controls. Red circles, mVSG genes; blue circles, BES VSG genes; black circle, VEX1; yellow circle, RBP6; grey circles, all genes.

The transition from epimastigote to metacyclic cells in the tsetse fly salivary gland results in the activation of MESs promoters and expression of *mVSG* genes (Graham and Barry 1995; Sharma et al., 2009). To determine the role of VEX1 in metacyclogenesis we established a doubly inducible TET ON system to simultaneously modulate *VEX1* expression levels and induce metacyclogenesis via *RBP6* overexpression (Supplementary Figure S1C). Initially we assessed the expression of two *mVSG* genes by RT-qPCR (Figure 5A; Table 3) at 4- and 8-days post induction along with *RBP6* and *VEX1*. In the parental cell line, with *RBP6* overexpression only, we see a 5-fold increase in *RBP6* expression and a 7–13 -fold increase in *mVSG397* and *mVSG653* expression (Figure 5A; Table 3). Strikingly, a reduction in *VEX1* resulted in poor *mVSG* expression as compared to the parental cell line (*RBP6* overexpression only), and surprisingly we also see a 6-fold reduction in *RBP6* expression (Figure 5A; Table 3). Conversely, when *VEX1* and *RBP6* are overexpressed, we see an 8-fold increase in *RBP6* expression and between 18–26-fold increase in *mVSG397* and *mVSG653* expression (Figure 5A; Table 3). We noted that the increase in *VEX1* overexpression cell line was subtle, but even in this context has a dramatic effect on both *mVSG* and *RBP6* expression levels (Figure 5A; Table 3).

**TABLE 3:** RT-qPCR values for RBP6 and mVSG expression Fold change and SD values for N = 3 for biological replicates.

	VEX1		RBP6		mVSG397		mVSG653	
	AVE	SD	AVE	SD	AVE	SD	AVE	SD
Parental D4	0.86	1.21	5.52	4.44	7.64	6.30	10.81	10.00
Parental D8	1.32	0.38	5.07	2.07	10.76	5.25	13.73	10.18
VEX1 overexpression D4	1.15	0.37	8.47	4.15	26.89	13.78	21.52	9.68
VEX1 overexpression D8	1.09	0.48	8.29	4.47	20.82	27.83	18.30	14.28
VEX1 RNAi D4	0.28	0.09	0.91	0.54	4.30	3.45	1.70	1.95
VEX1 RNAi D8	0.22	0.30	0.87	0.60	4.00	3.25	2.35	1.04

### VEX1 Positively Regulates *RBP6* and *mVSG* Expression During Metacyclogenesis

To further investigate the role of VEX1 in metacyclogenesis we wanted to see what global changes were associated with VEX1 RNAi or overexpression during metacyclogenesis. For this we performed transcriptomics analyses at day 4 and 8. Our analysis revealed that during metacyclogenesis, *VEX1* RNAi resulted in reduced *mVSG* expression and a concomitant reduction in the level of *RBP6* (Figure 5B) as we saw with the RT-qPCR (Figure 5A). This suggests that initiation of *mVSG* expression is crucial to this life cycle differentiating process. Using a pan-VSG antibody, we found that in cultures at day 4 and 8 cultures, no VSG expressing cells were seen (data not shown). We noted that there was a cohort of genes whose transcripts increased in abundance at day 4 and 8 (Figure 5B) following *RBP6* overexpression and *VEX1* RNAi. Analysis revealed a specific increase in abundance of silent BES linked genes, normally only expressed from the single active BES in bloodstream form cells (Supplementary Figure S4A). Differentiation from bloodstream form to procyclic form trypanosomes results in cessation of transcription of the *ESAG* and *VSG* genes from the active BES, however multiple BES promoters remain active a relatively equivalent level (Pays et al., 1989; Zomerdijk et al., 1990; Rudenko et al., 1994). Although BES protomers are active in procyclic cells, transcription terminates before the *ESAG7* gene (Zomerdijk et al., 1990; Rudenko et al., 1994). We then looked with more detail at which BES-linked genes were significantly upregulated, we found that *ESAG 10*, *7*, and *6* were 4 to 6-fold upregulated at all BESs, but this level of upregulation was not sustained (Supplementary Figure S4C, S5) suggesting transcription does not proceed across the whole BES. In fact, the BES-linked *VSG* genes were only on average 2-fold upregulated. We then looked at other Pol I transcribed genes and found that the procyclin associated genes *PAG1*, *PAG4*, and *PAG5* (Tb927.10.10240, Tb927.10.10210, Tb927.10.10230 respectively) showed an increase in transcript abundance (by 4, 6, 5-fold respectively on day 4) but not *EP1*, *EP2 Procyclin* or *GPEET* (Supplementary Figure S4A). This suggests that during metacyclogenesis, VEX1 is necessary for expression of *mVSG* genes and maintain silencing of BESs, but additional factors are required for full BES silencing in this life cycle stage. In contrast, following *VEX1* overexpression we see an increase in expression of *mVSG* genes and only moderate increase in BES linked *VSG* genes (Figure 5C), suggesting that VEX1 promotes *mVSG* expression. Unlike with *VEX1* RNAi, we see a more restrained increase in transcript abundance of BES linked genes and the procyclin and procyclin associated genes (Supplementary Figure S4B). Across the BES, *ESAG 10*, *7*, and *6* again showed the highest fold change, but by only 2-fold change on average, which was significantly lower than in the VEX1 RNAi (Supplementary Figure S4D, S5). Strikingly though, *mVSG* genes show an average of 6-fold increase in transcript abundance (Supplementary Figure S4D), revealing a positive role for VEX1 in *mVSG* transcription in metacyclic cells.

## Discussion

The developmental transitions that trypanosomes undergo as they cycle between the tsetse fly and mammalian host are coupled to dramatic changes in gene expression and especially in surface antigen expression. Central to trypanosomes survival in the mammalian host is the expression of a unique *VSG* gene that forms a dense protective barrier on the surface of the cell—both in the initial stages of the infection and once established. Our understanding of what leads to mammalian infectivity in African trypanosomes has long been limited by our ability to study key developmental transitions, however, this has changed with the establishment of the *RBP6* overexpression system (Kolev et al., 2012), and more recently with single-cell RNA sequencing of tsetse fly derived trypanosomes (Vigneron et al., 2020; Hutchinson et al., 2021). How these *VSG* genes are switched on in the tsetse fly salivary gland and the factors that control this process have remained unknown. Here we describe the role of VEX1 in the initiation of *mVSG* expression in metacyclic trypanosomes.

Localisation of VEX1 in the bloodstream form cells is intrinsically linked to function, with VEX1 accumulating at the single active BES and the spliced leader locus (Glover et al., 2016; Faria et al., 2019). The VEX complex undergoes a dramatic relocalisation from bloodstream form to insect stage procyclic form, potentially making the VEX complex available when *VSG* expression is reinitialised. Defining the localisation of VEX1 before and after metacyclogenesis is therefore key. Our *in vitro* and *in vivo* data show a relocalisation of VEX1 in insect stage cells associated with the expression of *mVSG* genes, suggesting that the VEX-complex may act similarly to that in bloodstream forms cells defining the single active MES. The variation in the number of VEX1 foci in metacyclic cells may represent the transition to single *mVSG* expression, but this remains to be shown.

Regulation of monoallelic *VSG* expression is lost when slender bloodstream forms differentiate into G_1_ arrested stumpy forms in the mammalian host (Amiguet-Vercher et al., 2004) and the VSG coat is shed in the tsetse fly (Roditi et al., 1989). In the procyclic form cell, *VSG* promoters are silenced and repositioned to the nuclear periphery (Navarro et al., 1999; Landeira and Navarro 2007). Depletion of the VEX complex in bloodstream form cells leads to loss of monoallelic *VSG* regulation (Glover et al., 2016; Faria et al., 2019) and here, we show that loss of VEX1 results in *VSG* expression in insect stage procyclic cells (Figures 1A,C). We observed a stronger effect on the MES which may be due to the difference in size between the BES and MES, with the former being up to 60 kb in length and the latter only 5 kb, and the processivity of the polymerase across these loci.

How *VSG* transcription, in either metacyclic or bloodstream form cells, is initiated is unclear. From their single cell RNA-sequencing of tsetse fly derived cells Hutchinson et al., 2021 proposed a “race model” for the initiation and establishment of monoallelic *mVSG* expression. They show a two-step process governs the establishment of monoallelic expression; 1) transcription is initiated at multiple MESs but 2) a single MES eventually dominates (Hutchinson et al., 2021). This process invokes the recruitment of VEX1 and VEX2 by the MESs which in turn triggers a positive feedback loop to recruit the splicing machinery, defines the ESB and drives transcription. The MES with the highest transcription level, recruiting most of the VEX-complex, and thereby depriving the other MESs, would outcompete the others and be established as the single active MES (Glover et al., 2016; Faria et al., 2021; Hutchinson et al., 2021). Several studies in bloodstream form cells also suggest a transcriptional race for establishment of monoallelic *VSG* expression. Firstly, although transcription is initiated at all BESs, it is only elongated over one (Kassem et al., 2014); secondly, during a forced transcriptional switch, where the active BES is switched off and a silent BES is activated, transcription transiently increased across multiple silent BESs in a suggested “probing” of silent *VSG*-ES before the cell fully activates one BES and undergoes a switching event (Aresta-Branco et al., 2016). Our data revealed that VEX1 influences initiation of *mVSG* expression. Where VEX1 is depleted, and *mVSG* expression is low, and metacyclogenesis fails (Figure 5). In fact, by increasing the abundance of VEX1, and presumably the VEX-complex during metacyclogenesis, not only does *mVSG* transcript abundance increase but so too does *RBP6* expression through a positive feedback loop (Figure 5).

In summary, we have shown that focal accumulation of VEX1 in the metacyclic trypanosome nucleus is dependent on the expression of a *VSG*, presumably defining the single active MES. Our findings have revealed that VEX1 modulates metacyclogenesis and that this life cycle differentiation step is dependent on the cell ability to initiate expression of *mVSG* genes.

## Data Availability

The datasets for VEX1 RNAi or overexpression in procyclic cells and VEX1 RNAi or overexpression in parallel with RBP6 overexpression in procyclic cells for this study can be found on the ENA PRJEB49957 (ERP134502).

## Supplementary Material

Supplementary Figure 1 Cell line set up. Protein and immunofluorescence analysis of VEX1^myc^ **(A)** RNAi and **(B)** overexpression. **(C)** Protein analysis of RBP6 overexpression and in conjunction with VEX1 overexpression or VEX1 RNAi.

Supplementary Figure 2 VEX1 localization in tsetse fly salivary gland-derived metacyclic cells. Immunofluorescence analysis of tsetse fly midgut-derived late procyclic / mesocyclic cells. The images correspond to maximal 3D projections of 0.24 μm stacks; scale bars 5 μm. N, nucleus; K, kinetoplast.

Supplementary Figure 3 VEX1 localization in Tsetse fly derived metacyclic cells. Immunofluorescence analysis of tsetse fly salivary gland derived late metacyclic cells. The images correspond to maximal 3D projections of 0.24 μm stacks; scale bars 5 μm. N, nucleus; K, kinetoplast.

Supplementary Figure 4 VEX1 RNAi silencing and overexpression results in upregulation of BES linked genes during metacyclogenesis. **(A)** RNA-seq analysis following RBP6 induction and VEX1 RNAi at 4- and 8-days post induction, values are averages from three independent RNAi strains relative to wild-type controls. **(B)** RNA-seq analysis following RBP6 induction and VEX1 overexpression at 4 and 8 days, values are averages from three independent VEX1 overexpression strains relative to wild-type controls. Blue circles, BES linked genes, Black circle, VEX1; Yellow circle, RBP6; Pink circle, Procyclin and Procyclin associated genes; Grey circles, all genes. **(C)** Upper panel: Schematic depicting a generic BES. Lower panel: Plot depicting the average fold change of BES linked genes following RBP6 overexpression and VEX1 RNAi. Error bar, SD. **(D)** Upper panel: Schematic depicting a generic BES. Lower panel: Plot depicting the average fold change of BES linked genes following RBP6 overexpression and VEX1 overexpression. Error bar, SD. Values are averages of three independent controls relative to wild-type control.

Supplementary Figure 5 VEX1 RNAi and overexpression modulate the level of *ESAG7* and *ESAG6* during metacyclogenesis. **(A)** Plot depicting the average of *ESAG 10*, *7*, and *6* between VEX1 overexpression and RNAi on day 4 **(B)** Plot depicting the average of *ESAG 10*, *7*, and *6* between VEX1 overexpression and RNAi on day 8 **(C)** Plot depicting the fold change of each BES linked *ESAG6* following RBP6 overexpression and VEX1 overexpression or RNAi. **(D)** Plot depicting the fold change of each BES linked *ESAG7* following RBP6 overexpression and VEX1 overexpression or RNAi. Error bar, SD. Significance was calculated using an unpaired t test; (*, P 0.03; ***, P, 0.0001; ****, P, < 0.0001).
