# Supplementary figures and images for: VEX1 Influences mVSG Expression During the Transition to Mammalian Infectivity in Trypanosoma brucei
Source: Front Cell Dev Biol. 2022 Apr 5;10:851475. doi: 10.3389/fcell.2022.851475 (PMC9017762; doi:10.3389/fcell.2022.851475)

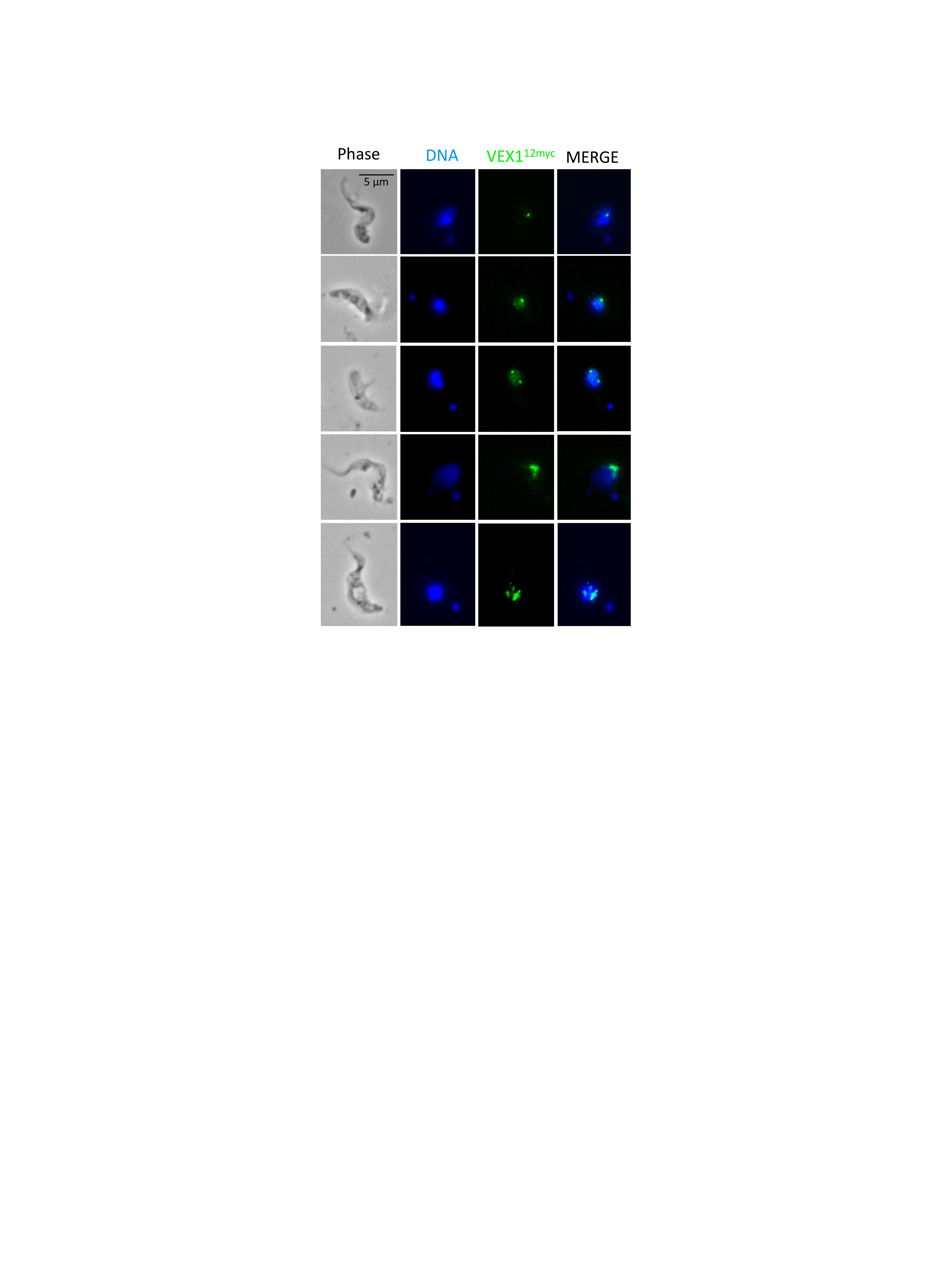

Supplement: Supplementary file 1 [file Image3.tiff]

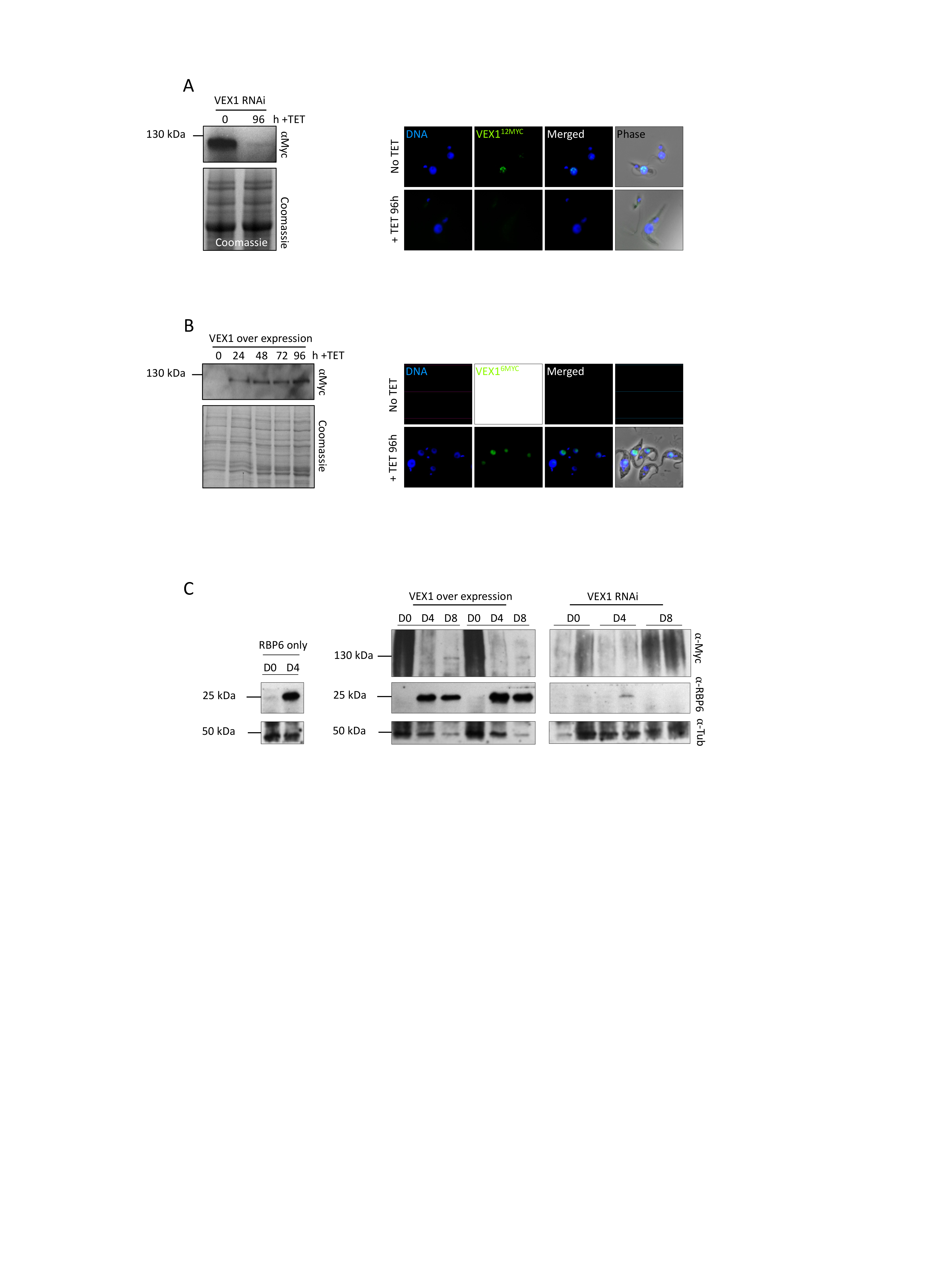

Supplement: Supplementary file 3 [file Image1.tiff]

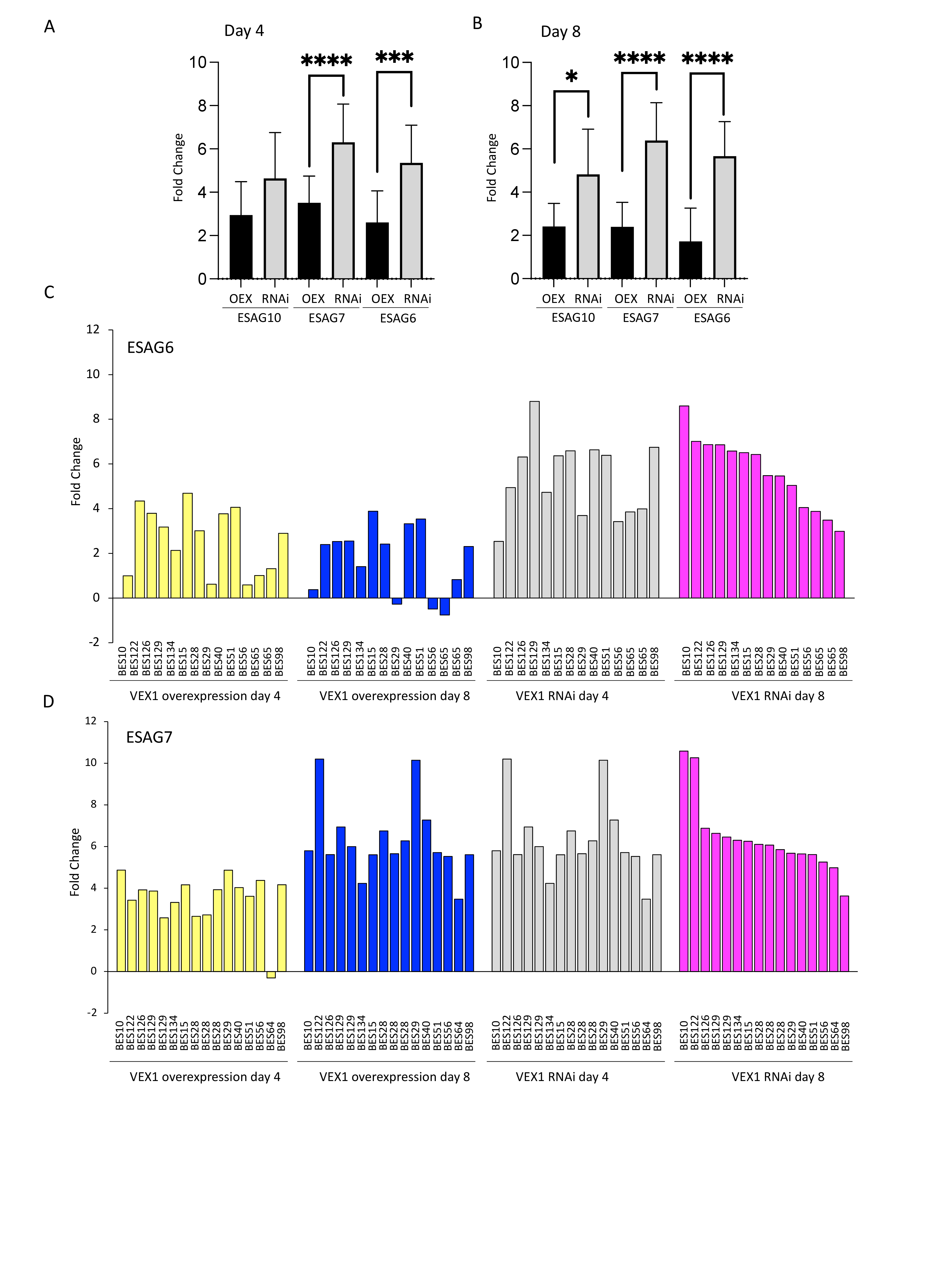

Supplement: Supplementary file 4 [file Image5.tiff]

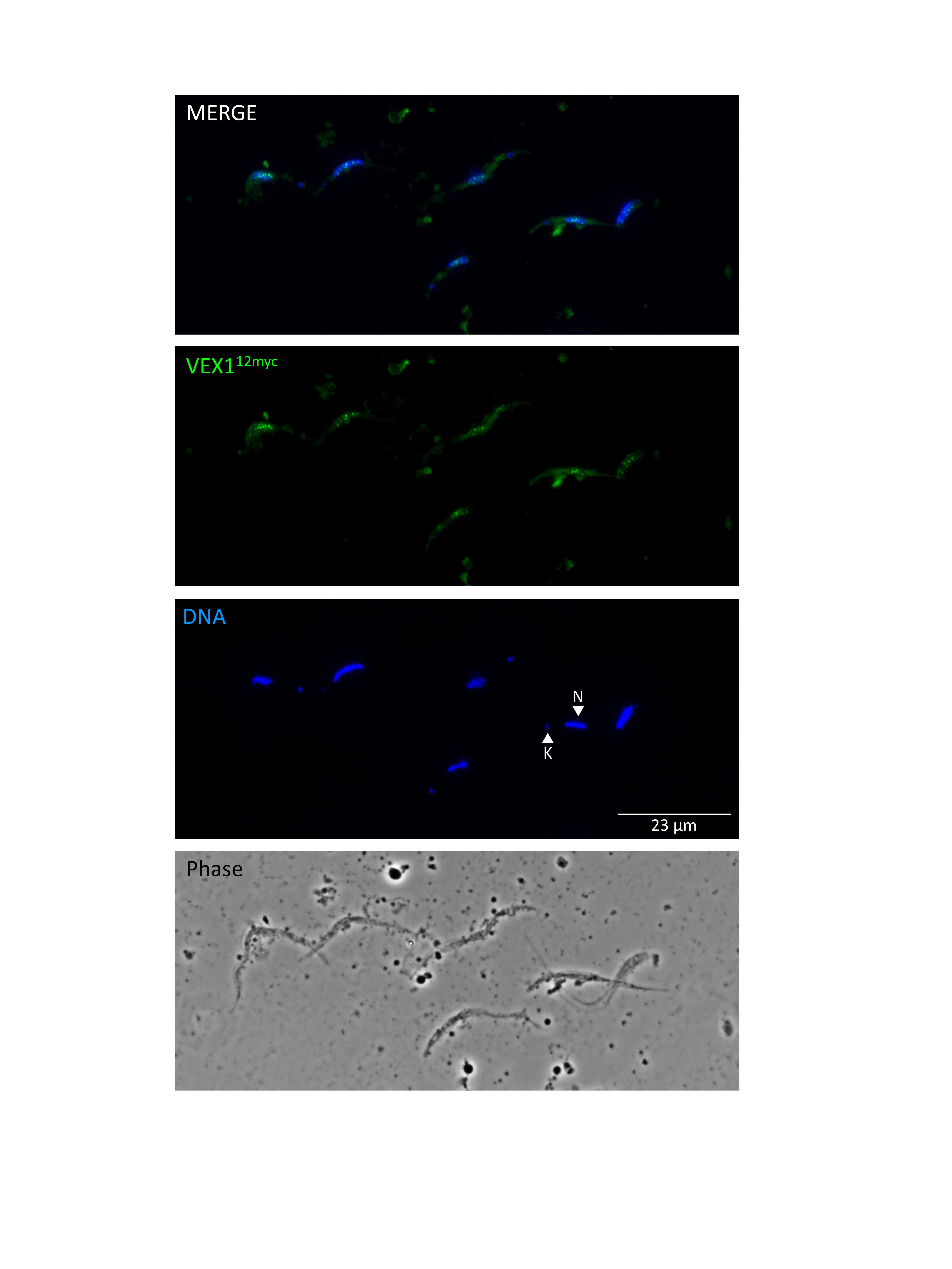

Supplement: Supplementary file 6 [file Image2.tiff]

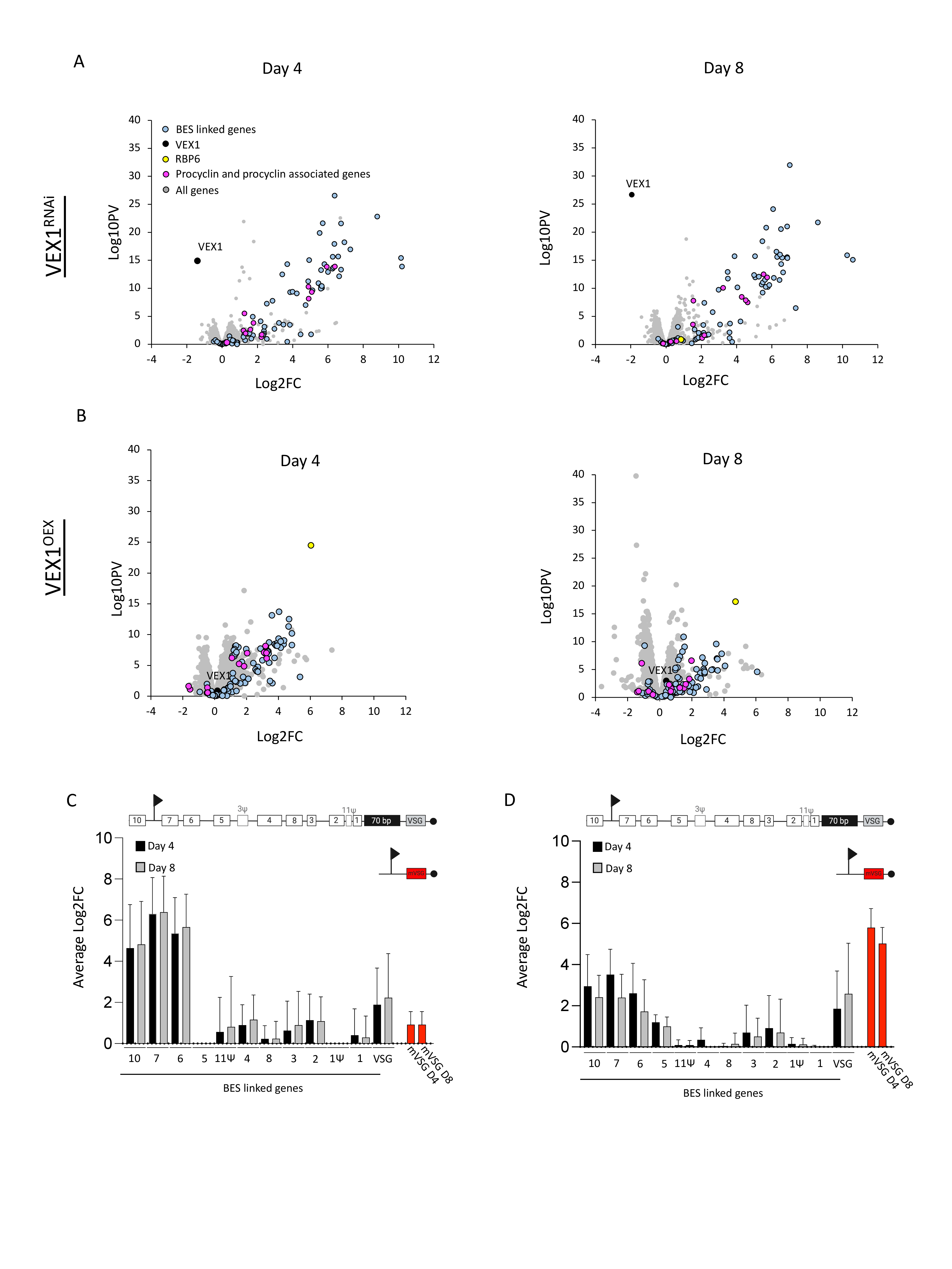

Supplement: Supplementary file 7 [file Image4.tiff]
